# Further validation of the THINC‐it tool and extension of the normative data set in a study of *n* = 10.019 typical controls

**DOI:** 10.1002/mpr.1922

**Published:** 2022-06-24

**Authors:** Maria Dalby, Peter Annas, 23andMe Research Team, John E. Harrison

**Affiliations:** ^1^ H. Lundbeck A/S Valby Denmark; ^2^ Department of Medical Epidemiology and Biostatistics Karolinska Institutet Stockholm Sweden; ^3^ 23andMe, Inc. Sunnyvale California USA; ^4^ Metis Cognition Ltd Warminster Wiltshire UK; ^5^ Institute of Psychiatry, Psychology and Neuroscience King's College London London UK; ^6^ Alzheimer Center AU Medical Center Amsterdam The Netherlands

**Keywords:** attention, cognition, computerised cognitive testing, depression, memory, norms, psychometric, THINC‐it

## Abstract

**Introduction:**

We report further validation and normative data for the THINC‐Integrated Tool (THINC‐it), a measure of cognitive function designed for use with individuals living with Major Depressive Disorder, but which is finding use in further psychiatric and neurological diseases. THINC‐it comprises four objective computerised cognitive tests based on traditional psychological paradigms and a version of the Perceived Deficits Questionnaire assessment.

**Methods:**

Sample size of *n* = 10.019 typical control study participants were tested on one to two occasions to further validate the reliability of THINC‐it. Temporal reliability was assessed across 120–180 days.

**Results:**

Test‐retest reliability correlations varied between *r* = 0.50 and 0.72 for the component measures and *r* = 0.75 (95% confidence intervals 0.74, 0.76) for the THINC‐it composite score. Normative data categorised by Age, Sex and Years of Education were calculated and the effect on task performance was reported.

**Discussion:**

Our analysis confirms previously reported levels of reliability and validates previously reported normative data values.

## 1 BACKGROUND

THINC‐Integrated Tool (THINC‐it) is composed of four computerised objective measures of cognitive function and a 5‐item version of the Perceived Deficits Questionnaire (PDQ‐5) which contains questions designed to elicit the study participants' view of their episodic memory, attentional skills and facets of executive function. The THINC‐it tool has been validated and reported as a method of detecting caseness in a cohort of individuals living with Major Depressive Disorder (MDD) (McIntyre et al., 2017). Further published work has reported validation of the assessment and its use to quantify the mediating effects of sleep quality, psychosocial function and pain (Cha et al., 2019, 2017a, 2017b; Harrison et al., 2018). Finally, the assessment is also currently being employed in clinical trials as an efficacy outcome measure (https://clinicaltrials.gov/ct2/results?cond=&term=THINC‐it, accessed March 9, 2020).

An early validation study (McIntyre et al., 2017) comparing the performance of typical controls with individuals living with MDD reported rates of caseness using THINC‐it data. Performance in the typical control cohort was also analysed to determine the key psychometric characteristics of the THINC‐it measures and to provide an initial estimate of normative performance (Harrison et al., 2018). However, the relatively modest size of the evaluated cohort (less than *n* = 100) provided only rudimentary norms with which to compare individual performance. We have recently obtained further normative data from the remote *Affective disorders, Environment, and Cognitive Trait (AFFECT) study* cohort of *n* = 23.836 typical controls, in which 10.019 had completed the THINC‐it test battery (Dalby et al., 2022). We report here the process of analysing this data to validate the instrument in a large, remote cohort and to expand the normative THINC‐it database. We reinvestigated THINC‐it's psychometric properties by further validity analysis, operationalised by the Coefficient of Variation (CoV), and temporal reliability calculating the test‐retest reliability (TRR).

## 2 METHODS

### 2.1 Study design

Typical control study participants were recruited from the 23andMe, Inc. database as part of the AFFECT study (Dalby et al., 2022). Participant eligibility criteria were: age between 18 and 50 years upon enrolment, residence in the United States, access to a desktop or laptop computer, and no reported diagnosis of Parkinson's disease, essential tremor, Schizophrenia, Alzheimer's disease, bipolar depression, major depression, generalized anxiety disorder, or PTSD as well as never having been prescribed an antidepressant, mood stabilizer, or antipsychotic medication. The study was conducted between August 2017 and November 2018.

### 2.2 Study participants

**FIGURE 1:**
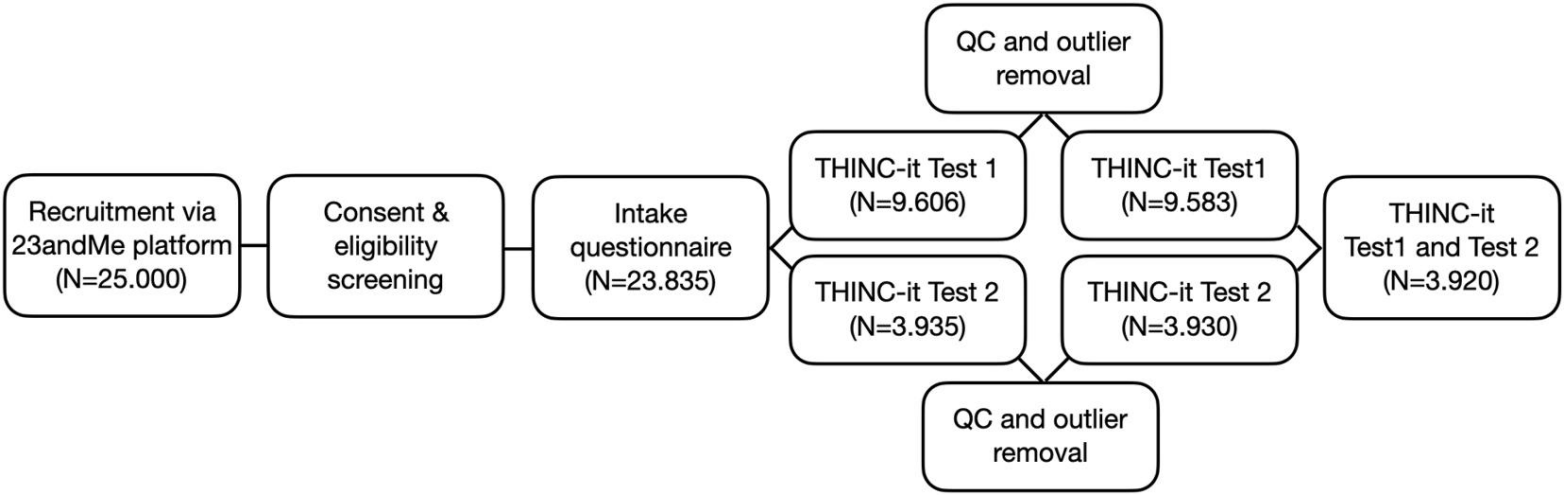
Study flow chart. The study flow was (1) recruitment by 23andMe Inc. user database, (2) consent to study criteria and meet control screening, (3) answer the intake baseline questionnaire remotely, as a minimum, (4) complete THINC‐it Test 1 after 2 months of study enrolment and/or complete THINC‐it test 2 after 7 months of study enrolment

In total, *n* = 23.836 participants were recruited into the study (see Figure 1 for details of study flow), 10,019 participants completed THINC‐it at least one time and 3920 participants completed the test twice. Full details of the cohort study are reported here (Dalby et al., 2022).

### 2.3 Assessments

THINC‐Integrated Tool tests were administered at least once (Figure 1). Study participants were invited to complete one follow‐up assessment at 6 months. As previously reported (Harrison et al., 2018) the measures that comprise THINC‐it were chosen according to prevailing best practice guidance for objective cognitive test selection (Ferris et al., 1997; Harrison & Maruff, 2008; Harrison and McIntyre, 2016). *Symbol Check* is a version of the One Back paradigm and is a test of working memory (Kirchner, 1958), and *Trails* is a version of the Trail Making Test Part B and assesses executive function (Lezak, 1995). *CodeBreaker* is based on the Digit Symbol Substitution Test a test that taps into a number of cognitive functions such as psychomotor speed, attention and executive function (Wechsler, 1939) and *Spotter* a version of a choice reaction time task and assesses attention (Donders, 1969).

A *z* score for each THINC‐it objective measure (i.e., Spotter, Symbol Check, CodeBreaker, Trails) was calculated referencing baseline performance as follow: [participant_*x*_ score on test_*y*_ − baseline mean on test_*y*_]/baseline standard deviation on test_*y*_. All *z* scores were sign‐adjusted, such that higher *z* scores denote better performance. A composite *z* score was calculated as the mean of *z* scores for all objective measures.

THINC‐Integrated Tool tests were specified for administration in the following order: The PDQ‐5, Spotter, Symbol Check, CodeBreaker, and Trails.

### 2.4 Quality control and outlier removal

Simple univariate statistics was applied for outlier removal by excluding primary outcome measures more than four standard deviations from the mean score. For the Trails objective measure, a time limit was set to 5 minutes and observations with no recorded game version was excluded. No filtering was applied to the PDQ‐5 assessment outcome.

We further required that study participants had completed, and QC passed a minimum of 3 out of four objective measures at each assessment time point.

### 2.5 Institutional Review Board (IRB)

Participants provided informed consent and participated in the research online, under a protocol approved by the external AAHRPP‐accredited IRB, Ethical and Independent Review Services (E&I Review). Participants were included in the analysis on the basis of consent status as checked at the time data analyses were initiated.

## 3 RESULTS

The baseline demographic of the study participants, including age, sex and educational level is presented in Table 1.

**TABLE 1:** Demographic details of study cohort

Characteristic (*N* = 9593)^a^	Mean (SD)	Number (%)
Gender		
Female		6247 (65.0%)
Male		3346 (35.0%)
Females age, years		
Age <30	32.3 (8.0)	2929 (46.9)
Age [31–40]		2179 (34.9)
Age >41		1139 (18.2)
Males age, years		
Age <30	33.7 (7.80)	1263 (37.7)
Age [31–40]		1368 (40.1)
Age >41		715 (21.4)
Education, years^b^		
Less than high‐school	16.1 (2.7)	25 (0.3)
High‐school diploma		377 (3.9)
Some college		973 (10.1)
Associates degree		440 (4.6)
Bachelors degree		3035 (31.6)
Masters degree		1689 (17.6)
Professional degree		779 (8.1)
Missing		2275 (23.7)

^a^ Based on controls who have completed at least 75% of all modules (i.e. 3) on at least one THINC‐it occurrence (*N* = 9593).

^b^ Map taxonomies of the highest educational to years of education using ISCED (Connelly et al., 2016).

**FIGURE 2:**
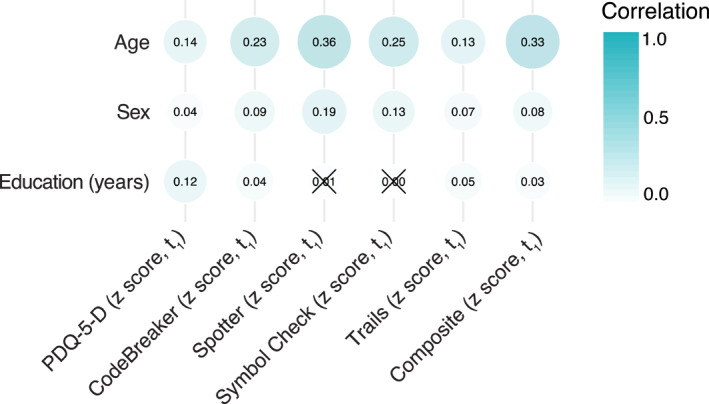
Normative data correlation. Spearman's correlation between; age, gender, years of education and THINC‐it performance from Assessment 1 (*n* = 9583). Primary task measures were Spotter (mean latency for correct responses, ms), CodeBreaker (number of correct responses), Symbol Check (number correct), Trails (time taken, ms), PDQ‐5‐D (score). Correlation estimates are given as numbers within each circle and by colour. Non‐significant correlation estimates are crossed over

Median and mean values with confidence intervals based on standard errors are reported for all THINC‐it tasks in Table 2. Partial mean performance (95% CI) and quantile (QT) scores for THINC‐it tests split by age and gender is reported in Table 3. To determine the possible impact of age and years of education on THINC‐it performance, we correlated these factors with the baseline THINC‐it composite score. A significant correlation of THINC‐it performance (i.e. composite score at baseline) with age (Spearman's *r* = −0.33, *p* < 2.2e^−16^), and a small but significant effect of years of education (Spearman's *r* = 0.03, *p* = 0.01) was observed. To test for possible effects of gender, THINC‐it baseline performance for males (*n* = 3346) was compared with that of females (*n* = 6237). Two independent samples *t*‐test yielded a significant higher performance in males (mean THINC‐it composite score = 0.07, SD = 0.68) than females (mean THINC‐it score = −0.04, SD = 0.72), (*p* = 2.2e^−15^). For primary task measures at baseline, the effect of age and sex was strongest on Spotter (Spearman's *r* = 0.36 and *r* = 0.19 respectively, *p* < 2.2e^−16^) and lowest for Trials (Spearman's *r* = 0.13 and *r* = 0.07 respectively, *p* < 1.3e^−11^) see Figure 2.

**TABLE 2:** Assessment 1 and 2 mean performance (95% CI) and quantile (QT) scores for THINC‐Integrated Tool (THINC‐it) tests

	Assessment 1^a^			Assessment 2^b^		
Task (primary measure)	1st, 3st QT	Median	Mean (95% CI)	1st, 3st QT	Median	Mean (95% CI)
Spotter (mean latency for correct responses, ms)	407, 523	456	494.7 (±3.1)	394, 497	438	466.4 (±4.0)
CodeBreaker (number of correct responses)	58, 75	66	66.7 (±0.3)	62, 79	70	70.7 (±0.4)
Symbol check (number correct)	25, 37	33	29.5 (±0.2)	31, 39	36	33.1 (±0.3)
Trails (time taken, ms)	17,102, 27,089	21,314	23,409 (±0.19)	15,789, 24,664	19,432	21,243 (±0.25)
PDQ‐5‐D (score)	7, 11	9	9.6 (±0.1)	7, 11	9	9.6 (±0.1)

^a^ based on an *n* = 9583.

^b^ based on an *n* = 3930.

**TABLE 3:** Partial mean performance (95% CI) and quantile (QT) scores for THINC‐Integrated Tool (THINC‐it) tests split by age and gender

Primary task measure	Assessment 1, mean (95% CI)^a^			Assessment 2, mean (95% CI)^b^		
	Age [18–30]	Age [31–40]	Age [41–50]	Age [18–30]	Age [31–40]	Age [41–50]
Spotter (mean latency for correct responses, ms)						
Male	423.72 (±4.77)	458.28 (±5.92)	538.67 (±13.22)	409.62 (±7.42)	437.13 (±7.83)	502.88 (±16.40)
Female	463.21 (±4.21)	520.28 (±6.58)	622.64 (±12.54)	436.23 (±4.90)	481.10 (±7.41)	589.38 (±19.02)
CodeBreaker (number of correct responses)						
Male	68.00 (±0.74)	64.95 (±0.67)	59.84 (±0.86)	72.60 (±1.19)	69.22 (±1.08)	63.72 (±1.32)
Female	70.27 (±0.50)	67.06 (±0.53)	61.63 (±0.72)	74.62 (±0.80)	71.31 (±0.82)	64.37 (±1.13)
Symbol check (number correct)						
Male	33.51 (±0.44)	31.20 (±0.50)	25.91 (±0.84)	36.36 (±0.54)	34.29 (±0.66)	30.55 (±1.16)
Female	30.73 (±0.33)	28.38 (±0.43)	23.79 (±0.67)	34.20 (±0.40)	32.23 (±0.57)	28.11 (±1.00)
Trails (time taken, s)						
Male	21.50 (±0.46)	22.50 (±0.46)	25.28 (±0.81)	19.35 (±0.63)	20.52 (±0.64)	21.93 (±0.92)
Female	22.61 (±0.30)	23.97 (±0.41)	26.54 (±0.64)	20.67 (±0.42)	21.88 (±0.55)	23.89 (±0.86)
PDQ‐5‐D (score)						
Male	9.83 (±0.18)	9.44 (±0.17)	8.82 (±0.24)	9.98 (±0.30)	9.30 (±0.27)	8.65 (±0.35)
Female	10.13 (±0.12)	9.54 (±0.14)	9.11 (±0.18)	10.11 (±0.19)	9.54 (±0.20)	9.14 (±0.2)

^a^ Based on an *n* = 9583.

^b^ Based on an *n* = 3930.

### 3.1 Reliability

The minimum time interval between assessments was 120 days and the maximum period 180 days. Temporal, or ‘test‐retest’, reliability was calculated by correlating Assessment 1 scores with the available Assessment 2 scores. This analysis yielded Spearman's correlation coefficients for the THINC‐it measures varying between 0.50 and 0.72 (all significant at *p* < 2.2e^−16^). These correlations are reported in Table 4, together with the CoV for each test, as well as difference between Assessment 1 and Assessment 2 expressed as an effect size (Cohen's *d*).

**TABLE 4:** Assessment 1 and 2 Coefficient of Variation (CoV), Test‐Retest Reliability (TRR) with confidence intervals (CI), and Cohen's *d* changes

Task (measure)	CoV (assessment 1)	CoV (assessment 2)	TRR (95% CI)^a^	Cohen's *d* difference^b^
Spotter (mean latency for correct responses, ms)	0.29	0.28	0.72 (±0.02)	0.14
CodeBreaker (number of correct responses)	0.19	0.19	0.67 (±0.02)	−0.23
Symbol check (number correct)	0.32	0.25	0.50 (±0.02)	−0.31
Trails (time taken, ms)	0.39	0.38	0.57 (±0.02)	0.15
THINC‐it composite	n/a	n/a	0.75 (±0.01)	−0.29
PDQ‐5‐D (score)	0.33	0.34	0.68 (±0.02)	−0.1

^a^ Based on an *n* = 3920.

^b^ Baseline as reference.

## 4 DISCUSSION

Published THINC‐it test data has provided evidence of the psychometric properties of the component measures, but only limited normative data. This further study has offered the opportunity to extend substantially the normative THINC‐it database, as well as to further investigate the temporal reliability of the included measures. Demographically, the study cohort reported in this study is comparable with that reported by Harrison et al., (2018). Mean age of this new cohort was 32.6 years versus 39.9, with a 58/42% F:M split in this study versus 65/35%. Years of Education was the same for both groups at 16 years. Temporal reliability was slightly reduced in the present study, with TRR scores ranging between 0.50 and 0.66 versus 0.74–0.81. However, the pattern of temporal reliability across tests was consistent, with Symbol Check exhibiting the lowest TRR in both studies and CodeBreaker the highest, again on both studies. The rank ordering of tests by TRR in both studies yields precisely the same order, suggesting continuity of the same underlying principles. The slightly reduced levels of TRR in the current study is likely a legacy of remote data collection. In the Harrison et al., study participants underwent supervised assessment, whereas in the current study, test completion was self‐administered. A further key difference is that in the Harrison et al. study follow‐up assessment was 1 week later, whereas in the current study the delay period was variable but required within 6 months of baseline assessment.

With respect to mean performance, scores in this study were in the case of CodeBreaker and Symbol Check virtually identical to the values reported by Harrison et al. (2018). Mean completion time for Trails was marginally quicker in the current study and 100 ms quicker for the Spotter task. Whereas for CodeBreaker and Symbol Check the key outcome metric is accuracy, that is, number correct, for both Trails and Spotter the key variable is response latency. One possible reason for the slightly quicker responses reported in the current study could be due to the precision of timing differences in the technology used. Support for this hypothesis is that the pattern of performance in both this and the Harrison et al. are consistent and that virtually identical mean scores were recorded in both studies for the two accuracy‐based assessments (CodeBreaker and Symbol Check). This pattern of data inclines us to the view that the current study results are valid and support the calculation of percentile scores reported in Table 3. This extension of the normative data addresses the issue of previous normative data analyses of THINC‐it performance being limited by the relatively small sample size for which data has been available. This new large dataset also permitted a more detailed review of the impact of age, education and sex.

It is our view that remote self‐administered cognitive assessment has considerable value in the context of large database studies such as the 23andMe/Lundbeck studies described here. The remote study set‐up enabled an extensive data collection in more than 10.000 typical control individuals and the availability of cognitive data extends considerably our understanding of the endophenotype of study participants in whom we are seeking to better comprehend the genetic contributions to the psychopathology of MDD. However, the utility of remote assessment extends to other contexts, including self‐administered cognitive assessment for general health purposes and the risk of developing disorders of cognition such as Alzheimer's disease, etc. Part of the motivation for developing THINC‐it for remote assessment purposes was to provide a brief assessment of key cognitive domains for recruiting study candidates for both exploratory and confirmatory clinical trials. The emergence of Covid‐19 infection and the worldwide epidemic that ensued has challenged us in conducting cognitive assessments. Our experience of employing THINC‐it in this current study suggests that the system may have utility when face‐to‐face assessment would be difficult or prohibited. In the context of the COVID‐19 pandemic, THINC‐it, as a free to use digital cognitive biomarker, offers opportunities to detect cognitive sequelae of infection. The availability of THINC‐it in fifteen languages, including Korean and Chinese versions, supports the prospects of use in large, multinational studies. In this context we note the publication of a recent account of successful employment of the THINC‐it system in Chinese‐speaking individuals (Hou et al., 2020).

### 4.1 Limitations of this study

The reported temporal reliability in this study is likely to be affected by the remote study setup and the long and variable delay period for the follow‐up assessment. The performance in remote versus supervised assessments have typically not been available in a comprehensive and detailed extent and needs future investigation. Furthermore, a typical study attrition as a consequence of remote study administration was also observed here.

## 5 CONCLUSIONS

Our extensive data collection and data analysis confirms previously reported levels of reliability and validates previous normative data values from age, gender and years of education. We here conclude that THINC‐it cognitive battery is a suitable tool for remote cognitive assessment in typical controls.

## CONFLICT OF INTEREST

Dr. Harrison reports receipt of personal fees in the past 2 years from Actinogen, AlzeCure, Aptinyx, Astra Zeneca, Athira Therapeutics, Axoltis, Axon Neuroscience, Axovant, Bial Biotech, Biogen Idec, BlackThornRx, Boehringer Ingelheim, Brands2life, Cerecin, Cognito, Cognition Therapeutics, Compass Pathways, Corlieve, Curasen, EIP Pharma, Eisai, G4X Discovery, GfHEU, Heptares, ImPACT, Ki Elements, LSP Operations, Lundbeck, Lysosome Therapeutics, MyCognition, Neurocentria, Neurocog, Neurodyn Inc, Neurotrack, the NHS, Novartis, Novo Nordisk, Nutricia, Probiodrug, Prothena, Recognify, Regeneron, reMYND, Rodin Therapeutics, Samumed, Sanofi, Signant, Syndesi Therapeutics, Takeda, Vivoryon Therapeutics and Winterlight Labs. Additionally, he holds stock options in Neurotrack Inc. and is a joint holder of patents with My Cognition Ltd. Drs Annas^,^ and Dalby^,^ are both employees of H. Lundbeck A/S.

## Data Availability

The variant‐level data for the 23andMe dataset are fully disclosed in the manuscript. Individual‐level data are not publicly available due to participant confidentiality, and in accordance with the IRB‐approved protocol under which the study was conducted.
